# Deciphering the Skin Anti-Aging and Hair Growth Promoting Mechanisms of *Opophytum forskahlii* Seed Oil via Network Pharmacology

**DOI:** 10.3390/ijms27010277

**Published:** 2025-12-26

**Authors:** Shaimaa R. Ahmed, Hanan Khojah, Maram Aldera, Jenan Alsarah, Dai Alwaghid, Luluh Hamdan, Hadeel Aljuwair, Manal Alshammari, Hanadi Albalawi, Reema Aldekhail, Abdullah Alazmi, Sumera Qasim

**Affiliations:** 1 Department of Pharmacognosy, College of Pharmacy, Jouf University, Sakaka 72341, Aljouf, Saudi Arabia; 2 Department of Pharmacognosy, College of Pharmacy, Nursing and Medical Sciences, Riyadh Elm University, Riyadh 12734, Riyadh, Saudi Arabia; hanan.khojah@riyadh.edu.sa; 3 College of Pharmacy, Jouf University, Sakaka 72341, Aljouf, Saudi Arabia; mramaldera@gmail.com (M.A.); jenan7s15@gmail.com (J.A.); daialkhalde@gmail.com (D.A.); 421209407@ju.edu.sa (L.H.); hadee.ii99e@gmail.com (H.A.); manal.03450@gmail.com (M.A.); hanadialbalawi44@gmail.com (H.A.); 4 College of Medicine, King Saud University, Riyadh 11472, Riyadh, Saudi Arabia; reema.aldekhail@gmail.com; 5 College of Medicine, Jouf University, Sakaka 72388, Aljouf, Saudi Arabia; dr.alazmiabdullah@gmail.com; 6 Department of Pharmacology, College of Pharmacy, Jouf University, Sakaka 72341, Aljouf, Saudi Arabia; qsumera@ju.edu.sa

**Keywords:** *Opophytum forsskalii*, phytochemicals, anti-aging, hair growth, network pharmacology

## Abstract

*Opophytum forskahlii* has a well-established ethnopharmacological significance. This study aimed to assess the skin anti-aging and hair growth-promoting activities of *O. forskahlii* seed oil (OFSO) and the underlying mechanism. GC-MS profiling revealed high levels of unsaturated fatty acids, linoleic acid (55.46%), and oleic acid (38.54%). The skin anti-aging activity of OFSO (3.125–100 µg/mL) was evaluated in normal human dermal fibroblasts (NHDFs) using MTT and enzyme inhibition assays. OFSO was non-cytotoxic and enhanced fibroblast proliferation in a dose-dependent manner, reaching 145.5% of control at 100 µg/mL (*p* < 0.05). OFSO significantly (*p* < 0.05) inhibited collagenase (48%), hyaluronidase (53%), elastase (57%), and tyrosinase (55%). The oil showed anti-inflammatory activity by inhibiting COX-1 and COX-2 (0.01–100 µg/mL) with IC_50_ = 0.125 and 0.014 µg/mL, respectively. The hair growth promoting efficacy was assessed using adult male Wistar rats, randomly divided into control, OFSO-treated, and 2% minoxidil-treated groups (5 rats/group). Hair growth was assessed through visual scoring over 14 days of topical application and confirmed by histological examination and hair follicle counting. On day 14, the OFSO-treated group displayed almost complete hair coverage (score about 5.0), exceeding minoxidil (about 4.0), and significantly increased hair follicle number (14.0 ± 1 vs. 9.2 ± 0.8, *p* < 0.05). Histology confirmed that OFSO promoted hair follicle growth, differentiation, and transition from the telogen to the anagen phase. Network pharmacology analysis, integrating targets predicted via SwissTargetPrediction and disease-associated genes from GeneCards, identified PPARG, ESR1, and IL6 as key hub genes underlying OFSO’s effects. PPARG enhances antioxidant defenses, anti-inflammatory responses, and sebaceous gland function; ESR1 supports collagen production, skin elasticity, and follicle vascularization; and IL6 modulates inflammation and triggers the anagen phase of hair growth. Functional enrichment revealed modulation of PPAR, estrogen, prolactin, and arachidonic acid metabolism pathways, suggesting that OFSO may regulate lipid metabolism, inflammation, hormonal signaling, and tissue regeneration. OFSO demonstrated promising anti-aging and hair growth activities, supporting further development and testing of cosmetic formulations.

## 1. Introduction

The growing demand for natural remedies in cosmetic formulations led to more studies into new plant-based sources with diverse biological activities [1]. Skin and hair are the main means of communication with the outside world. Through complex biological processes like oxidative stress, inflammation, and disrupted follicular cycles, skin and hair are constantly exposed to an array of risk factors that contribute to aging and hair loss. A growing preference for natural alternatives and concerns over adverse effects of synthetic drugs have led scientists to refocus their attention on little-studied medicinal plants [2]. It is also increasingly recognized that skincare and haircare share similar biological targets, such as antioxidant defense mechanisms and modulation of inflammatory cytokines [3].

*Opophytum forskahlii* Hochst ex Boiss (Aizoaceae) is a succulent species, commonly called Samh in Arabic culture [4]. The plant is indigenous to arid climates of Arabian countries, for example, Egypt, Jordan, Kuwait, Qatar, Yemen and Saudi Arabia [4,5]. The seeds have long served as a nutritional resource for Bedouin communities inhabiting difficult desert environments [6]. Recently, several studies have revealed several bioactivities of *O. forskahlii*, including antidiabetic, anti-ulcer, antimicrobial, and antioxidant effects [7,8,9,10,11,12,13], pointing to potential value in functional foods and therapeutic application.

Plant-derived oils have long been incorporated into cosmetic formulations because of their capacity to form an occlusive or semi-occlusive film that protects the skin barrier. Beyond their physical effects, several oils, such as almond, jojoba, soybean, and avocado, exhibit distinct anti-inflammatory, antioxidant, and barrier-restorative actions that benefit both skin and hair follicle physiology [14]. As topical agents, plant seed oils provide advantages because they combine emollient and skin-repairing lipid phytochemicals.

Previous research on Samh seed oil revealed a high concentration of fatty acids, phytosterols, and lipophilic constituents. The oil showed significant antioxidant and antifungal properties, including inhibition of fungal species commonly found in human hair [12]. According to the chemical profile and bioactivity data, *O. forsskalii* seed oil (OFSO) is a promising natural ingredient for cosmetic applications that aim to delay skin aging and promote hair growth.

Network pharmacology has become a powerful tool for deciphering complex biological interactions between phytochemicals and biological pathways. The tool is shifting from a one-drug-one-target model to an integrated multicomponent-multitarget one [15,16]. Using this tool, it is possible to propose mechanistic hypotheses explaining observed experimental results by building compound-target-pathway networks. Applying network pharmacology to OFSO allows the identification of key molecular targets and signaling pathways associated with its dermatological effects [16].

Despite the nutritional and medicinal relevance of *O. forsskalii*, the dermatological potential of its seed remains unexplored.

The present study combined GC-MS profiling, in vitro skin anti-aging assays, in vivo hair-growth evaluation, and network pharmacology analysis to investigate the dermatological potential of *O. forsskalii* seed oil (OFSO). By integrating chemical, experimental, and computational approaches, we aimed to clarify the biological activities and underlying mechanisms of OFSO and to provide a scientific basis for its possible use in cosmetic and dermatological formulations.

## 2. Results

### 2.1. GC-MS Profiling of OFSO

The chemical composition of OFSO was determined by GC-MS analysis (Figure S1). Four compounds were identified (Table 1), predominantly unsaturated fatty acids: linoleic acid was the most abundant (55.46%), followed by oleic acid (38.54%), palmitic acid (5.58%), and a trace of cyclopropanebutanoic acid (0.41%).

### 2.2. Skin Antiaging Potential

#### 2.2.1. Effect of OFSO on NHDFs Cell Viability

**Figure 1:**
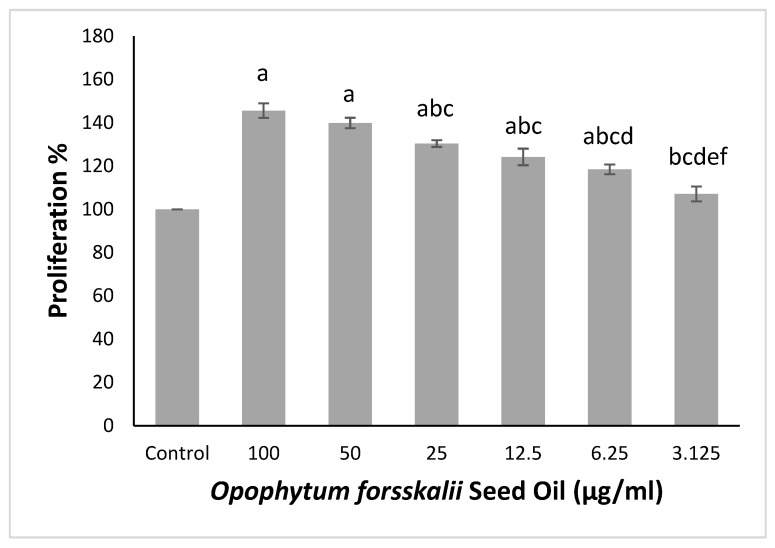
Effect of OFSO on normal human dermal fibroblast cell viability using MTT assay. Data are presented as mean ± SD (*n* = 3). Data were analyzed using one-way analysis of variance (ANOVA) followed by Bonferroni’s multiple comparison post hoc test. Different letters (a–f) indicate significant differences between groups (*p* < 0.05).

OSFO significantly increased normal human dermal fibroblast proliferation dose-dependently (Figure 1). The highest proliferation was observed at 100 µg/mL (145.5%), followed closely by 50 µg/mL (139.9%). Moderate increases in proliferation occurred at 25 µg/mL (130.4%) and 12.5 µg/mL (124.1%). Lower concentrations (6.25 and 3.125 µg/mL) continued to enhance proliferation (108.4% and 107.1%, respectively).

#### 2.2.2. Inhibition of Skin Aging-Related Enzymes

**Figure 2:**
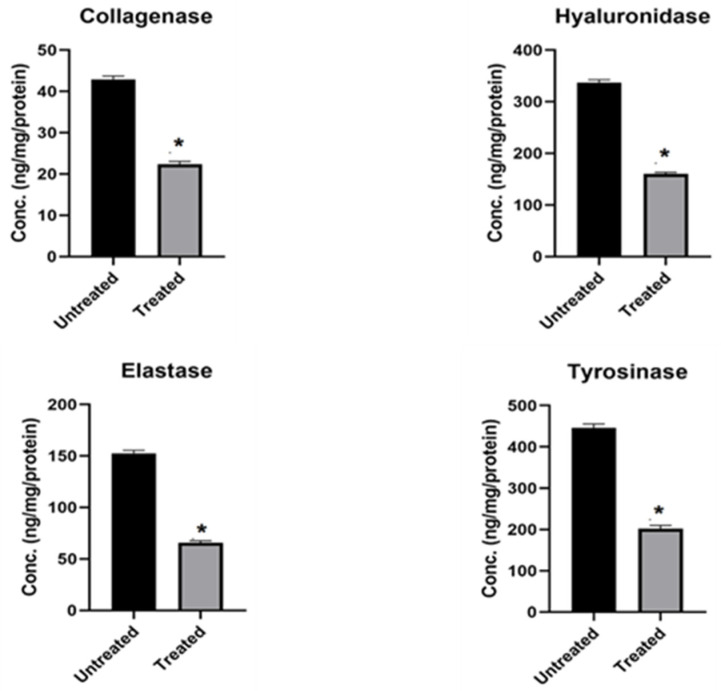
Effect of OFSO on skin aging-related enzymes activities (collagenase, hyaluronidase, elastase, and tyrosinase). Data are presented as mean ± SE (*n* = 3). An independent samples *t*-test was used to compare the untreated and treated human fibroblast cell lines. * *p* < 0.05.

Normal human dermal fibroblasts treated with OFSO demonstrated a significant decrease in collagenase, hyaluronidase, elastase, and tyrosinase protein concentrations (*p* < 0.0001), as shown in Figure 2.

Collagenase levels decreased from 42.91 ± 0.840 ng/mg/protein to 22.37 ± 0.682 ng/mg/protein (48% inhibition) following OFSO treatment. Hyaluronidase levels dropped from 337.3 ± 5.074 ng/mg/protein to 160.1 ± 3.210 ng/mg/protein (53% inhibition). Elastase levels also reduced from 152.6 ± 2.746 ng/mg/protein to 65.61 ± 1.982 ng/mg/protein (57% inhibition. Finally, tyrosinase concentration decreased from 446.2 ± 9.565 ng/mg/protein to 201.7 ± 8.338 ng/mg/protein (55% inhibition).

#### 2.2.3. Inhibition of COX1 and COX2 Activity

OFSO showed dose-dependent suppression of COX1 and COX2 activity, with IC_50_ values of 0.125 ± 0.0008 µg/mL for COX1 and 0.014 ± 0.0046 µg/mL for COX2.

### 2.3. Effects of OFSO on Hair Growth in Rats

#### 2.3.1. Gross Evaluation of Hair Growth

**Figure 3:**
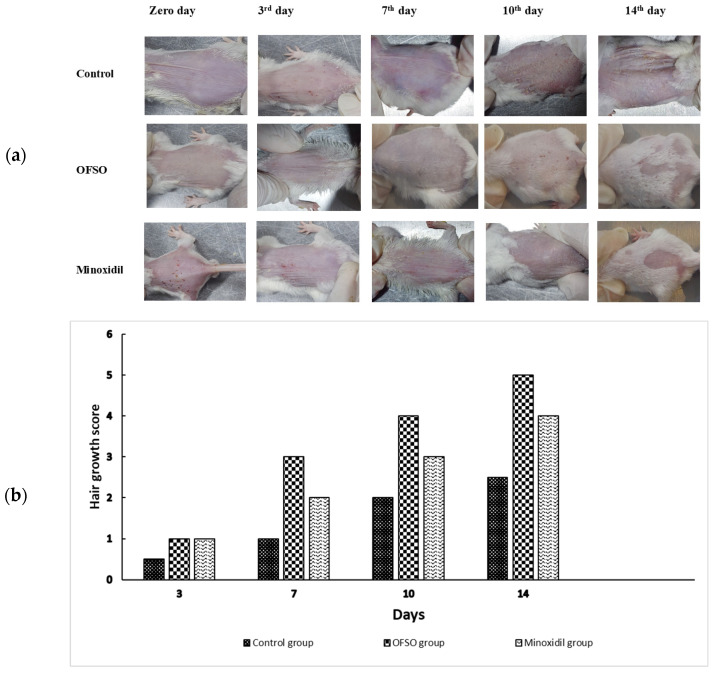
Effect of OFSO on hair growth in adult male Wistar rats (*n* = 5): (**a**) Digital images showing progressive hair growth in rats over 14 days of treatment. Photographs were taken on days 0, 3, 7, 10, and 14; (**b**) Hair growth score (*p* < 0.05).

Topical treatment with OFSO accelerated hair growth throughout the 14-day period compared to control and minoxidil treated groups (Figure 3a,b). At the start (day 0), all groups showed complete depilation. By day 3, both the minoxidil and OFSO groups had early hair sprouts (hair growth scores about 1.0), whereas the control group barely changed (score remaining at 0.5).

Rats treated with OFSO showed denser follicular activation on day seven (growth score near 3.0), which was higher than that of the minoxidil group (near 2.0). By day 10, the minoxidil group had reached 3.0 and the OFSO group had more hair coverage (score about 4.0), while the control group remained at 2.0. On day 14, OFSO-treated group displayed almost complete hair coverage (score about 5.0), surpassing both minoxidil (about 4.0) and control (about 2.5). All of these findings show that topical OFSO is superior to the reference drug minoxidil in terms of speeding up the onset and intensity of hair growth.

#### 2.3.2. Histopathological Findings

**Figure 4:**
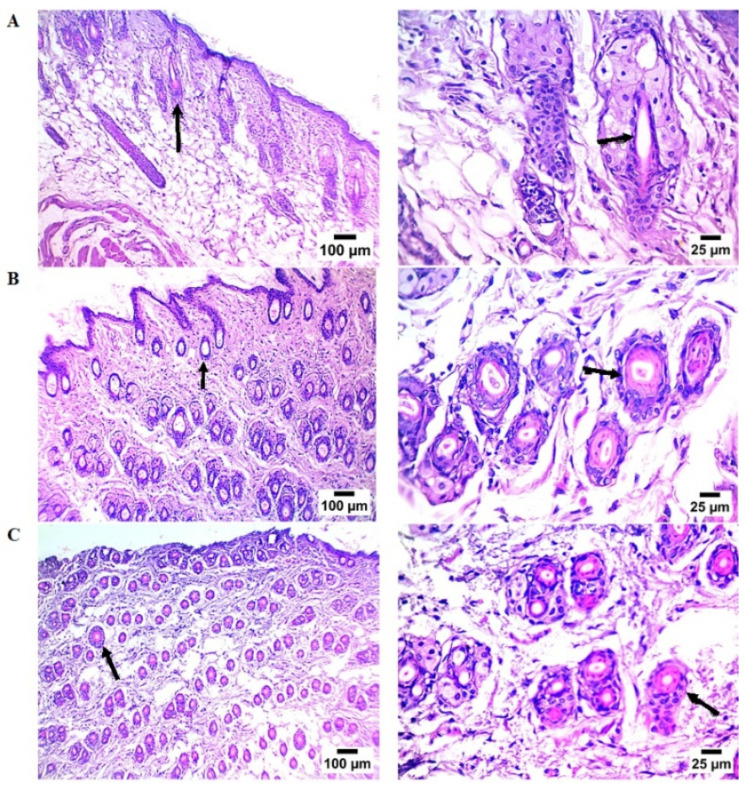
Photomicrographs of dorsal skin sections from different treatment groups after 14 days, stained with hematoxylin and eosin (H&E). (**A**) control, (**B**) OFSO; (**C**) Minoxidil. Black arrows indicate hair follicle generation in the dermis.

The control group showed low follicular density and little anagen activity (Figure 4A). Keratinocyte growth decreased, and the epidermis was thin and irregular. The dermis showed few, unevenly spaced hair follicles, many in the telogen (resting) phase, appearing tiny, shallow, and with atrophic bulbs.

Conversely, the OFSO-treated group (Figure 4B) displayed thicker epidermis and improved cellular organization. There are many active hair follicles in the dermis, which are tightly packed and extend deeper into the dermal layers. The majority of follicles are in the anagen (growth) phase, exhibiting noticeable hair shafts, big bulbs, and well-developed papillae. The minoxidil-treated group (Figure 4C) showed a dermis densely packed with recently formed hair follicles. These follicles’ morphology and depth were similar to those in the OFSO group, suggesting a strong anagen phase induction.

The mean number of hair follicles in dorsal skin sections is reported in Table 2. Both the OFSO-treated group and the minoxidil group showed a significant increase in hair follicle count compared to the control group. The OFSO-treated group showed a marked increase (14.0 ± 1) in hair follicle number. The topical application of minoxidil demonstrated significant stimulation in hair follicle count (9.2 ± 0.80). OFSO produced the highest follicle count, significantly superior to the minoxidil group.

### 2.4. Network Pharmacology Results

#### 2.4.1. Predicted Target Genes

SwissTarget Prediction yielded a total of 183 target genes for the four identified compounds (Table S1). Thirty-one genes overlapped with skin antiaging-related genes (Table S2) while ninety-six genes overlapped with hair growth promotion-related genes (Table S3). These overlapping genes were used for further interaction and enrichment analysis.

#### 2.4.2. PPI Network and Hub Gene Identification

**Figure 5:**
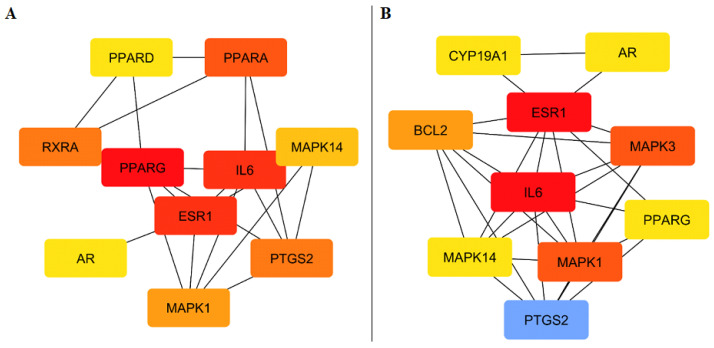
Top ten target genes involved in: (**A**) Skin antiaging; (**B**) hair growth promotion activities. Red nodes indicate highly connected hub genes, orange nodes represent moderately connected genes, yellow nodes indicate genes with low connectivity, and blue nodes represent peripheral or less-connected targets.

According to PPI network analysis, the key hub genes associated with skin anti-aging activity (Figure 5A) were PPARG (10), IL6 (9), ESR1 (9), PPARA (8), MAPK1 (8), MAPK14 (8), PTGS2 (8), RXRA (7), PPARD (7), and AR (7). While for hair growth (Figure 5B) the major hub genes were IL6 (15), ESR1 (15), PTGS2 (15), MAPK3 (10), MAPK1 (10), BCL2 (9), AR (8), PPARG (8), MAPK14 (8), and CYP19A1 (8).

#### 2.4.3. GO Enrichment Analysis

**Figure 6:**
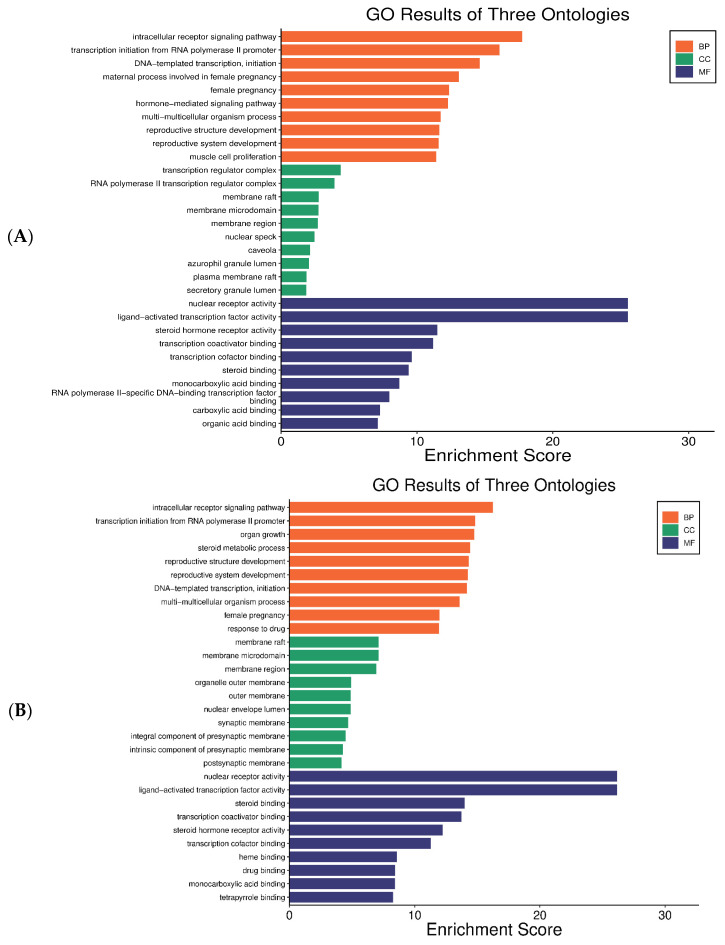
Gene Ontology (GO) enrichment analysis of the target genes: (**A**) Skin antiaging; (**B**) hair growth promotion. BP (Biological process, orange), MF (molecular function, blue) and CC (cellular component, green).

Gene Ontology (GO) enrichment analysis classified the anti-aging targets into biological process (BP), cellular component (CC), and molecular function (MF) categories (Figure 6A). Key BP terms included intracellular receptor signaling, RNA polymerase II–dependent transcription initiation, DNA-templated transcription, hormone-mediated signaling, and reproductive structure development. Enrichment in the CC category, such as transcription regulator complex, membrane raft, nuclear speck, and secretory granule lumen, indicates the subcellular localization of active signaling molecules. MF enrichment highlighted nuclear receptor activity, ligand-activated transcription factor activity, steroid hormone receptor activity, and transcription coactivator binding. Collectively, these results suggest that the anti-aging targets are mainly involved in transcriptional regulation and hormone-dependent signaling pathways.

Similarly, GO enrichment of the 96 hair growth-related targets (Figure 6B) identified BP terms such as intracellular receptor signaling and steroid metabolic processes, both of which influence androgen and estrogen pathways central to hair follicle cycling and androgenetic alopecia. MF terms included cytokine activity, enzyme binding, and steroid hormone receptor activity, functions essential for hormonal signaling and inflammation control in hair follicles. CC enrichment in the plasma membrane, cytosol, and extracellular region reflects active signal transduction and interactions within the dermal papilla microenvironment.

#### 2.4.4. KEGG Pathway Analysis

**Figure 7:**
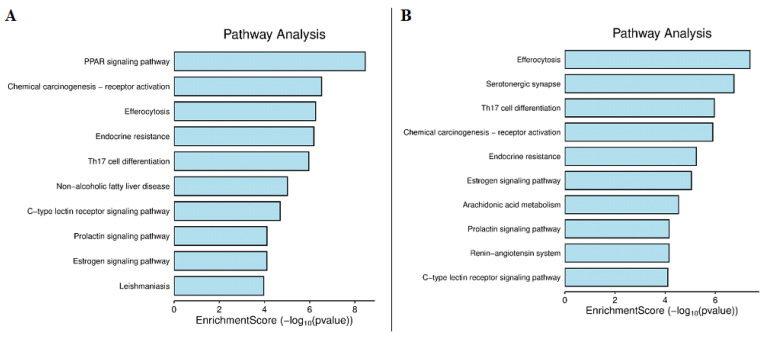
KEGG enrichment analysis of target genes: (**A**) Skin antiaging; (**B**) hair growth promotion.

KEGG pathway enrichment analysis identified various biological pathways connected with anti-aging targets (Figure 7A). PPAR signaling pathway was the most enriched pathway, followed by chemical carcinogenesis-receptor activation, efferocytosis, endocrine resistance, and Th17 cell differentiation. Additional pathways, including non-alcoholic fatty liver disease (NAFLD), C-type lectin receptor signaling, prolactin signaling, estrogen signaling, and leishmaniasis, were also enriched. The predominance of these pathways indicates that the identified targets are primarily involved in lipid metabolism, inflammatory modulation, hormone regulation, and cellular stress responses, all of which are closely linked to skin aging mechanisms.

For hair growth–related targets (Figure 7B), several enriched pathways were directly connected to follicular biology (Figure 7B). Estrogen signaling pathway plays a protective role in hair follicle maintenance. Alterations in this pathway can lead to disrupted follicle cycling and hair thinning. Arachidonic acid metabolism produces pro-inflammatory eicosanoids, which are implicated in scalp inflammation and hair follicle regression during the catagen phase. Prolactin signaling pathway is also relevant, as prolactin receptors are expressed in hair follicles, and prolactin dysregulation has been associated with telogen effluvium and other hair growth disturbances.

## 3. Discussion

The present study demonstrates that *Opophytum forskahlii* Seed Oil (OFSO) is rich in bioactive unsaturated fatty acids and possesses significant anti-aging and hair growth promoting activities. GC-MS analysis identified linoleic and oleic acids as major constituents, consistent with previously published data [12]. The high linoleic acid content (55%) in OFSO aligns with reports indicating its role in skin health. Linoleic acid contributes to skin anti-aging by restoring and maintaining the lipid barrier of the stratum corneum through the formation of acyl-ceramides, as well as by modulating inflammation and keratinocyte differentiation. Furthermore, biochemical analyses have demonstrated that linoleic acid levels decrease in aged skin, which may contribute to barrier dysfunction and visible signs of skin aging [17]. Similarly, numerous oleic acid rich oils are efficient penetration and barrier-repairing agents that lessen photoaging symptoms [14,18]. On the other hand, previous studies showed linoleic acid increases hair follicle dermal papilla cell proliferation and upregulates hair growth factor expression (VEGF, IGF-1, KGF, HGF) and activates Wnt signaling pathway for promoting hair growth [19,20]. The formerly described benefits of linoleic acid and oleic acid verify their contribution to OFSO’s skin anti-aging and hair growth promoting effects.

The observed increase in dermal fibroblast proliferation supports OFSO’s antiaging potential. Human dermal fibroblasts are the main producer of the extracellular matrix (ECM) components, including collagen, elastin, and glycosaminoglycans (such as hyaluronic acid). These elements are essential for maintaining the skin’s hydration, elasticity, and structural integrity [21,22,23]. So, one of the main targets in the formulation of antiaging treatment is agents that stimulate fibroblast proliferation. In line with previous studies, the results that OFSO is non-cytotoxic to NHDFs and significantly boost fibroblast proliferation supports its potential anti-aging effects. The oil may help preserve skin structure and function by promoting dermal regeneration and supporting ECM maintenance.

In addition, OFSO significantly inhibited key enzymes involved in extracellular matrix degradation and melanogenesis. These findings are consistent with previous reports that highlight enzyme inhibition as a central strategy for mitigating skin aging [24,25].

OFSO also showed selective COX2 inhibition, indicating anti-inflammatory properties. Inflammation is a well-recognized contributor to skin aging and impaired tissue repair. Consistent with previous research, OFSO’s anti-inflammatory activity may justify its use to delay the inflammatory-related changes associated with skin aging [26].

Beyond its skin anti-aging potential, OFSO exhibited robust hair growth promoting activity. Topical application accelerated the onset of hair growth and increased follicular density more effectively than the positive control minoxidil. Histological evaluation further confirmed that OFSO increased the growth and differentiation of hair follicles, accelerated their transition from the telogen (resting) phase to the anagen (growth) phase. Probably, this effect is due to the bioactive fatty acids contained in the oil (linoleic and oleic acids) [19,27]. Similar improvement in follicular density and anagen induction have been observed with other fatty acid rich oils, which activate Wnt/β-catenin signaling and hair growth factors [19,20,28].

Network pharmacology analysis provided insights into the molecular mechanism underlying these findings. PPARG, ESR1, and IL6 emerged as key hub genes, indicating their key regulatory roles in skin and hair health. PPARG supports antioxidant defenses and anti-inflammatory mechanisms essential for maintaining skin barrier integrity [29]. Sebaceous gland activity and keratinocyte differentiation, both essential for maintaining the scalp barrier and good follicle structure, are influenced by PPARG [30]. Likewise, the prominent position of ESR1 is consistent with its established involvement in collagen production, skin elasticity, and protection against oxidative damage [31]. By regulating estrogenic activity and vascular supply to hair follicles, the ESR1 gene appears to promote hair follicle regeneration [32]. IL6, a pro-inflammatory cytokine frequently elevated during skin aging, acts as a major mediator of age-related inflammatory processes [33]. Importantly, the anagen phase of hair follicle growth is triggered by IL6 [34]. Taken together, these findings suggest that the coordinated actions of PPARG, ESR1, and IL6 could serve as promising molecular targets of OFSO for counteracting skin aging hair loss disorders.

GO and KEGG enrichment analyses revealed that OFSO exerts its effects through several interconnected pathways. Anti-aging targets enriched in PPAR signaling, underlining its role in lipid balance and protection against oxidative stress. On the other hand, hair growth effects were linked to estrogen and prolactin signaling. This proves the importance of hormonal regulation in maintaining healthy follicle development. Furthermore, pathways such as arachidonic acid metabolism and Th17 cell differentiation indicate that OFSO can modulate immune and inflammatory responses. This may protect the skin and hair follicles from inflammation-induced aging and hair loss.

Despite the promising results, some limitations should be considered. First, the in vitro assays using fibroblasts and enzymes cannot fully replicate the complexity of human skin physiology. Second, although network pharmacology analysis provides a valuable predication of key targets and pathways, remains computational require validation through in vitro and in vivo experiments.

In conclusion, OFSO demonstrates significant potential as a natural agent for skin anti-aging and hair growth promotion through modulation of inflammation, oxidative stress, and hormone-mediated signaling, driven by unsaturated fatty acids. These findings highlight OFSO as a promising natural cosmeceutical component with potential applications in both anti-aging skincare and hair growth formulations.

## 4. Materials and Methods

### 4.1. Plant Material and Preparation of Oil

Seeds of *Opophytum forskahlii* Hochst. ex Boiss. were purchased in May 2024 as a powdered product from AlMeqdaa market, a local brand based in Sakaka, Al-jouf, Saudi Arabia. Powdered seeds (500 g) were extracted with 2.5 L of n-hexane as the extraction solvent. The mixture was subjected to ultrasound-assisted extraction using an ultrasonic bath (power Sonic 410 ultrasonic cleaner, Hwashin Technology Co., Ltd.; Seoul, Republic of Korea) for 45 min. Then, the mixture was filtered through Whatman No. 1 filter paper, and the filtrate was concentrated using a rotary evaporator (Buchi Rotavapor R-210, Flawil, Switzerland) at 40 °C, yielding the crude seed oil (10 mL). The obtained oil was stored in airtight amber glass bottles at 4 °C until further use in chemical profiling and biological evaluation.

### 4.2. Chemicals

Human fibroblast cells were obtained from American Type Culture Collection. Dulbecco’ modified Eagle’s medium (DMEM) medium, fetal bovine serum (FBS), and trypsin solution were purchased from Gibco (Grand Island, NY, USA). 3-(4,5-dimethylthiazol-2-yl)-2,5-diphenyl-2H-tetrazolium bromide (MTT) was obtained from Sigma-Aldrich (St. Louis, MO, USA). Human Collagenase I (Cat. No. MBS261969), human hyaluronidase (Haase, Cat. No. MBS727688), human elastase (ELA, Cat. No. MBS729999), human tyrosinase (TYR, Cat. No. MBS720969) ELISA kits were obtained from MyBioSource, San Diego, CA, USA. RayBio^®^ Quantichrom™ COX-1 (Cat. No. COX1S-100) and COX-2 (Cat. No. COX2S-100) inhibitor screening kit were purchased from RayBiotech Life Inc. (Peachtree Corners, GA, USA). All other chemicals and reagents used in the current study were of analytical grade and were provided by Sigma-Aldrich Chemical Co. (Arklow, Ireland).

### 4.3. GC-MS Profiling of the Seed Oil

Fatty acid methyl esters (FAMEs) were prepared from OFSO using an alkali-catalyzed reaction using methanol and 2 M potassium hydroxide, then injected into hexane. The resulting FAME analysis was carried out using a GC-MS system (Agilent Technologies, Santa Clara, CA, USA) at the Central Laboratories Network, National Research Centre, Cairo, Egypt. The system is equipped with a gas chromatograph (7890B) coupled to a mass spectrometer detector (5977A). Chromatographic separation was achieved on HP-5MS column (30 m × 0.25 mm i.d., 0.25 μm film thickness). Hydrogen was used as the carrier gas at a constant flow rate of 3.0 mL/min and a splitless injection volume of 1 µL. The temperature program was as follows: 40 °C for 1 min; rise at 10 °C/min to 200 °C and hold for 1 min; rise at 20 °C/min to 220 °C and hold for 1 min; then rise at 30 °C/min to 320 °C and hold for 10 min. The injector and detector temperatures were kept at 250 °C and 320 °C, respectively. Mass spectra were obtained using electron ionization (EI) at 70 eV, with a scan range of *m*/*z* 50–800 and a solvent delay of 3.7 min. The mass temperature was 230 °C, while Quad was 150 °C. Different constituents were identified by comparing their fragmentation patterns to those retrieved from Wiley and NIST Mass Spectral Library data.

### 4.4. Skin Anti-Aging Potential

#### 4.4.1. Cell Culture and Treatment

Normal human dermal fibroblasts (NHDFs) were cultured in Dulbecco’s Modified Eagle Medium (DMEM) supplemented with 10% fetal bovine serum (FBS) and 1% penicillin-streptomycin in a humidified incubator with 5% CO_2_ at 37 °C. Cells were seeded in 96-well or appropriate culture plates and allowed to adhere. After 24 h, cells were treated with OFSO at predetermined concentrations, while untreated cells served as controls.

#### 4.4.2. Cell Viability (MTT Assay)

Cell viability and cytotoxicity were assessed using the MTT assay. NHDFs were seeded in a 96-well tissue culture plate at a density of 1 × 10^5^ cells/mL (100 µL/well) and incubated at 37 °C with 5% CO_2_ for 24 h. After incubation, the growth medium was removed, and the cell monolayer was washed twice with fresh medium. A sample of 100 µL of OFSO at concentrations (3.125, 6.25, 12.5, 25, 50, and 100 μg/mL) was added to the cells and incubated for 24 h. Maintenance medium was only applied to control wells. The plates were kept at 37 °C and monitored for morphological changes as a sign of cytotoxicity. Following treatment, 20 µL of MTT solution (5 mg/mL in PBS) was added to each well, and the plates were shaken for 5 min at 150 rpm to ensure proper mixing. Formazan crystals were then formed by incubating the samples at 37 °C for 4 h with 5% CO_2_. The optical density (OD) of the samples was measured at 560 nm using a microplate reader. Cell viability was calculated as a percentage relative to control wells.

#### 4.4.3. Modulation of Skin Aging-Related Enzymes

The levels of collagenase, hyaluronidase, elastase, and tyrosinase in treated and untreated fibroblast cell lines were measured in order to assess the impact of OFSO on cellular aging. Protein extracts were made after cells were harvested following treatment. According to the manufacturer’s instructions, enzyme concentrations were measured using commercially available ELISA kits. Results were expressed as ng/mg of each protein. Every experiment was carried out in triplicate, and the mean ± SEM was used to present the data. An independent sample *t*-test was used to determine the statistical significance between treated and untreated cells. *p*-values < 0.05 was considered statistically significant.

#### 4.4.4. Cyclooxygenase (COX1 and COX2) Inhibition Assays

The anti-inflammatory potential of OFSO was evaluated by measuring COX1 and COX2 activities. Enzymes inhibition was assessed using the RayBiotech/Quantichrom COX inhibitor screening kit according to the manufacturer’s instructions. Recombinant COX1 and COX2 enzymes were pre-incubated with different concentrations of OFSO (0.01, 0.1, 1, 10, and 100 µg/mL) dissolved in DMSO, ensuring a final DMSO concentration ≤ 1% (*v*/*v*). The reaction was initiated by adding arachidonic acid as the substrate and incubated at 37 °C for 10 min. The COX reaction product was measured colorimetrically at 450 nm using a BIOLINE ELISA (Delhi, India) reader. All assays were performed in triplicate, and results are presented as mean ± SEM. Percent inhibition was calculated relative to vehicle-treated control wells. IC_50_ values (concentration required to inhibit 50% of enzyme activity) were calculated from dose–response curves using the Quest Graph™ IC_50_ Calculator, (web-based tool, 2025 version) [35].

### 4.5. Hair Growth Promoting Activity

#### 4.5.1. Animals

Fifteen adult male Wistar rats weighing 180–200 g were obtained from the animal house of Nahda University, Beni-Suef, Egypt. Rats were kept in an air-conditioned (25 ± 1 °C) pathogen-controlled animal experimental room to acclimatize for two weeks for adaptation before being subjected to laboratory experiments, with free access to standard forage and tap water ad libitum. All experimental procedures were approved by the Ethical Committee of Nahda University following the guide for the care and use of laboratory animal (No. NUB-022-024).

#### 4.5.2. Experimental Design

Hair removal was performed as described by Magalhaes et al. [36]. Rats were anesthetized with thiopental sodium (40 mg/kg, i.p., 2.5% solution) prior to depilation. The dorsal area was initially shaved using an electric hair shaver (Philips, s1121/40, Amsterdam, The Netherlands), followed by the application of cold wax strips (Veet Minima Strips, Reckitt Benckiser, Slough, UK). A broader region (3 cm × 2 cm) was shaved, while waxing was restricted to a smaller area (2 cm × 1.5 cm) to avoid wax contact with unshaved skin. Wax strips were applied once on the stretched skin and swiftly removed against the direction of hair growth.

The animals were randomly divided into three groups (five rats in each): control, OFSO-treated, and 2% minoxidil-treated. Each group received a once-daily topical application of either normal saline (control), 0.5 mL of OFSO, or 0.5 mL of standard 2% minoxidil solution to the designated dorsal area. Hair growth in the treated regions was monitored, and digital photographs were taken on days 0, 3, 7, 10 and 14 using a Sony FX 2 digital camera^®^ with a 1.3× magnification lens. Gross hair growth was assessed by visual scoring as follows: 0 = no hair growth (the skin is entirely smooth), 1 = 20% hair growth (initial emergence of hair), 2 = 20–40% hair growth (short hair partially covering the area), 3 = 40–60% hair growth (predominantly covered area with visible soft hair), 4 = 60–80% hair growth (densely covered, resembling typical fur), 5 = 80–100% hair growth (area fully covered with dense hair).

#### 4.5.3. Histopathological Study

After day 14, the animals were slaughtered, and the skin was removed for histological examination, as described by Bancroft et al. [37]. Skin samples from various rat groups were fixed in 10% formalin saline for 24 h, then rinsed with water and dehydrated using a series of alcohol solutions. The specimens were then cleaned in xylene, embedded in paraffin beeswax blocks, and stored at 56 °C for 24 h. A microtome (Leica Microsystems SM2400, Cambridge, UK) was used to cut 4–6 µm thick sections from paraffin blocks. The sections were then deparaffinized and stained with hematoxylin and eosin (H&E, Sigma-Aldrich, St. Louis, MO, USA). The sections were examined under a light microscope (Olympus, Melville, NY, USA) for histological evaluation and hair follicle count to confirm macroscopic results.

### 4.6. Statistical Analysis

Data were analyzed by one-way ANOVA followed by Post Hoc Tukey for multiple comparisons. The results are expressed as mean ± SD, and statistical significance was accepted for *p* < 0.05.

### 4.7. Network Pharmacology Study

#### 4.7.1. Target Gene Prediction

Four constituents (palmitic acid, linoleic acid, oleic acid, and cyclopropanebutanoic acid) were identified in OFSO. To predict potential human protein targets for each compound, the SwissTargetPrediction database (https://www.swisstargetprediction.ch/ (accessed on 3 July 2025)) was employed. Predictions were conducted using the Homo sapiens model, and only targets with a probability score above the threshold were retained for further analysis.

#### 4.7.2. Collection of Disease-Associated Genes

Genes associated with antiaging and hair growth promotion were retrieved from the GeneCards database (https://www.genecards.org/ (accessed on 3 July 2025)) by searching the keywords “skin aging and hair loss.” Genes were filtered based on their relevance scores to ensure the inclusion of those with strong associations to the condition.

#### 4.7.3. Identification of Overlapping Genes

The predicted targets of OFSO constituents were cross-referenced with the antiaging and hair growth promotion-related gene set to identify overlapping genes. These shared genes were considered putative molecular targets involved in the therapeutic effect of OFSO as antiaging and hair growth promotor.

#### 4.7.4. Protein–Protein Interaction (PPI) Network Construction and Hub Gene Analysis

The overlapping gene set was uploaded to the STRING database (https://string-db.org/ (accessed on 3 July 2025)) for construction of a protein–protein interaction (PPI) network, using a high confidence interaction score (≥0.7) to ensure robustness of the network. The resulting interaction data were visualized and analyzed using Cytoscape software (v3.9.1). Key hub genes were identified using the CytoHubba plugin (version 0.1) within Cytoscape software (version 3.9.1), applying degree centrality as the ranking criterion. 

#### 4.7.5. Gene Ontology (GO) and KEGG Pathway Enrichment Analysis

Functional enrichment analysis of the overlapping genes was performed using the DAVID Bioinformatics Resources (https://david.ncifcrf.gov/ (accessed on 3 July 2025)). Gene Ontology (GO) terms were categorized into biological processes (BP), molecular functions (MF), and cellular components (CC). Additionally, Kyoto Encyclopedia of Genes and Genomes (KEGG) pathway enrichment analysis was conducted to identify relevant signaling pathways. A *p*-value of <0.05 was considered statistically significant.

## 5. Conclusions

This study conducted a thorough assessment of *Opophytum forskahlii* seed oil (OFSO) utilizing chemical, biological, and computational approaches. GC-MS profiling identified linoleic and oleic acids as the main active constituents. In vitro assays confirmed its safety and significant anti-aging efficacy through inhibition of collagenase, elastase, hyaluronidase, and tyrosinase enzymes. In vivo study showed marked improvement of hair growth, indicated by increased follicle density and histological improvement. Network pharmacology provides molecular evidence by revealing that OFSO’s active compounds modulate inflammation, oxidative stress, and hormone-mediated signaling, three interrelated processes central to skin regeneration and follicular vitality. These findings highlight OFSO as a promising natural cosmeceutical component with potential applications in both anti-aging skincare and hair growth formulations. Future clinical studies are essential to validate its therapeutic efficacy in humans, thereby endorsing its utilization in cosmeceutical formulations.

## Figures and Tables

**Table 1:** Chemical composition of *Opophytum forskahlii* seed oil (OFSO) analyzed by GC-MS.

Peak	Rt * (min.)	Component	Formula	Percentage (%)
1	14.76	Hexadecanoic acid, methyl ester	C_17_H_34_O_2_	5.58
2	16.328	9,12-Octadecadienoic acid (Z,Z)-, methyl ester (Linoleic acid)	C_19_H_34_O_2_	55.46
3	16.387	9-Octadecenoic acid (Z)-, methyl ester (Oleic acid)	C_19_H_36_O_2_	38.54
4	16.714	Cyclopropanebutanoic acid, 2-[[2-[[2-[(2-pentylcyclopropyl)methyl]cyclopropyl]methyl]cyclopropyl]methyl]-, methyl ester	C_25_H_42_O_2_	0.41

* Retention time.

**Table 2:** Hair follicle count in adult male Wistar rats following topical application of OFSO and minoxidil.

Groups	Hair Follicle Count *
Control	3.6 ± 0.93
OFSO	14.0 ± 1.00 ^a^
Minoxidil	9.2 ± 0.80 ^a,b^

* Values are mean ± SD (*n* = 5). Data were analyzed by one-way ANOVA followed by Post Hoc Tukkey for multiple comparisons. ^a^ significant difference vs. control. ^b^ Significant difference vs. OFSO at (*p* < 0.05).

## Data Availability

The raw data supporting the conclusions of this article are not publicly available due to their large size and because some raw files require proprietary software for proper visualization. Data are available from the corresponding author upon request.

## Supplementary Materials

The following supporting information can be downloaded at: https://www.mdpi.com/article/10.3390/ijms27010277/s1.

## Abbreviations

The following abbreviations are used in this manuscript:

COX1	Cyclooxygenase 1
COX2	Cyclooxygenase 2
ESR1	Estrogen Receptor 1
GC-MS	Gas chromatography-mass spectrometry
GO	Gene Ontology
IL6	Interleukin 6
KEGG	Kyoto Encyclopedia of Genes and Genomes
NHDFs	normal human dermal fibroblasts
PPARG	Peroxisome Proliferator-Activated Receptor Gamma.
