# Supplementary material for: Deciphering the Skin Anti-Aging and Hair Growth Promoting Mechanisms of Opophytum forskahlii Seed Oil via Network Pharmacology
Source: Int J Mol Sci. 2025 Dec 26;27(1):277. doi: 10.3390/ijms27010277 (PMC12785290; doi:10.3390/ijms27010277)
Supplement: Supplementary file 1 [file ijms-27-00277-s001.zip › ijms-4058508-supplementary.pdf]

Supplementary material

Deciphering the Skin Anti-Aging and Hair Growth Promoting Mechanisms of *Opophytum forskahlii* Seed Oil via Network Pharmacology

Shaimaa R. Ahmed [1,*], Hanan Khojah [2], Maram Aldera [3], Jenan Alsarah [3], Dai Alwaghid [3], Luluh Hamdan [3],
Hadeel Aljuwair [3], Manal Alshammari [3], Hanadi Albalawi [3], Reema Aldekhail [4], Abdullah Alazmi [5] and Sumera Qasim [6]

1 Department of Pharmacognosy, College of Pharmacy, Jouf University, Aljouf, Sakaka 72341, Saudi Arabia, srmorsi@ju.edu.sa
2 Department of Pharmacognosy, College of Pharmacy, Nursing and Medical Sciences, Riyadh Elm
University, Riyadh, Riyadh 12734, Saudi Arabia; hanan.khojah@riyadh.edu.sa
3 College of Pharmacy, Jouf University, Aljouf, Sakaka 72341, Saudi Arabia; mramaldera@gmail.com (M.A.); jenan7s15@gmail.com (J.A.); daialkhalde@gmail.com (D.A.); 421209407@ju.edu.sa (L.H.); hadee.ii99e@gmail.com (H.A.); manal.03450@gmail.com (M.A.); hanadialbalawi44@gmail.com (H.A.)
4 College of Medicine, King Saud University, Riyadh, Riyadh 11472, Saudi Arabia; reema.aldekhail@gmail.com
5 College of Medicine, Jouf University, Sakaka, Jouf 72388, Saudi Arabia; dr.alazmiabdullah@gmail.com
6 Department of Pharmacology, College of Pharmacy, Jouf University, Aljouf, Sakaka 72341, Saudi Arabia; qsumera@ju.edu.sa
* Correspondence: srmorsi@ju.edu.sa; Tel.: +966-562-019-622

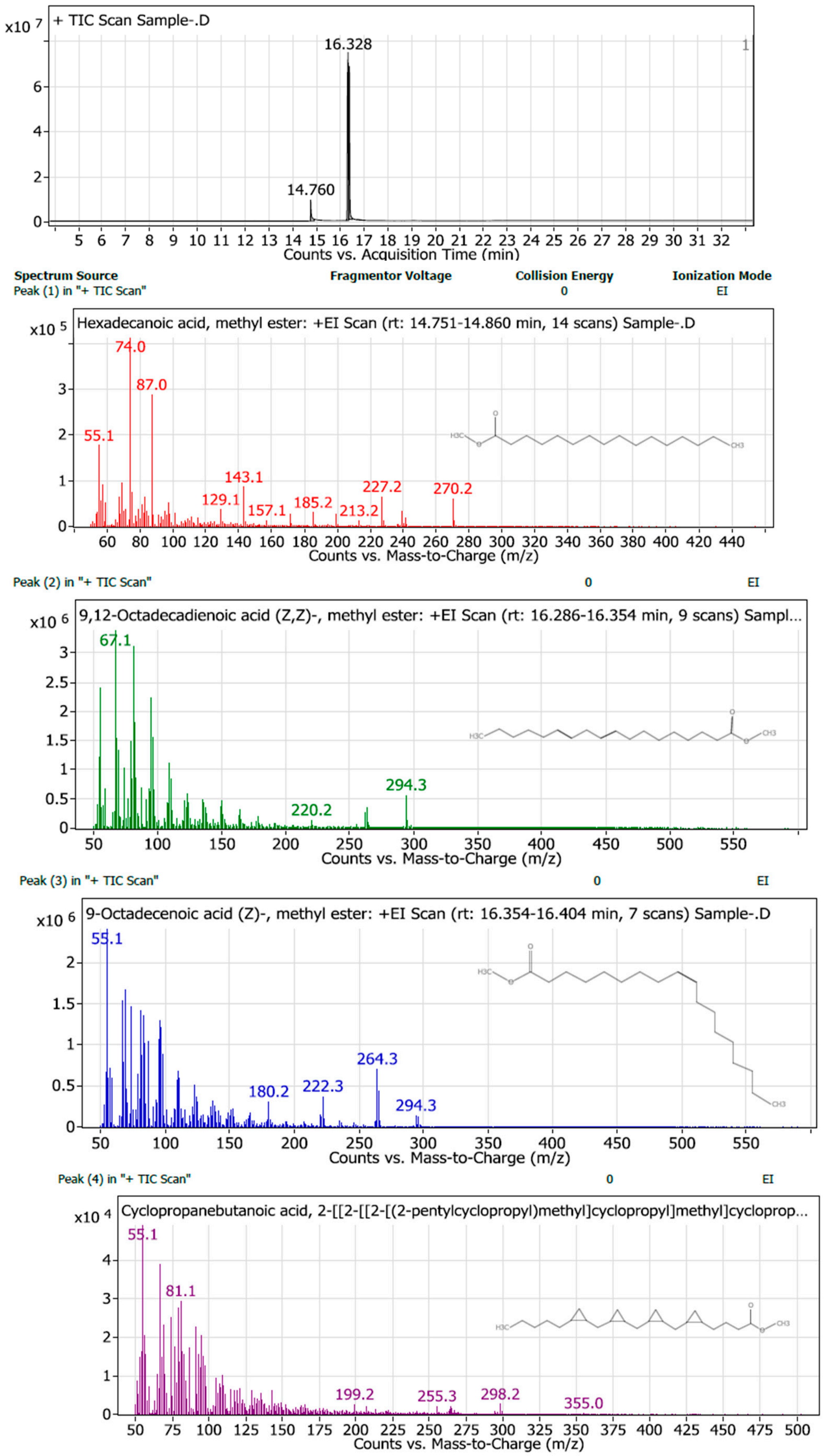

Figure S1: GC-MS analysis of *Opophytum forsskalii* seed oil (OFSO)

Table S1: Target genes for identified compounds in OFSO

Genes				
FABP4	UBA2	GRM8	HTR2B+A134:A177	GLRA1
FABP3	GRM5	CPB2	ADRA2B	EPHX2
FABP5	PTGES	GRM4	MAPK14	ABCC1
PPARD	FDFT1	RNPEP	MAPK1	ABCB1
FFAR1	AKR1B1	MAOA	IL6	SAE1
FABP2	KMO	AHR	GLUL	RORB
SLC22A6	HDAC3	TOP2A	PTPN11	ITGAL
NR1H4	HMGCR	PTGS1	PLA2G4A	SAE1
PPARA	HCAR2	CDC25C	EDNRA	
AR	FAAH	PREP	MAPK3	
VDR	PPARG	CHRNA7	MDM2	
POLB	TERT	SHH	CMA1	
CDC25A	FABP1	CNR1	CTSG	
GPBAR1	CPT1A	ALOX5	PSEN2	
HSD11B2	PTGS2	LTB4R	CCKBR	
PHF8	SLC1A2	NR3C1	ENPP2	
KDM5C	GRM2	TOP1	HNF4A	
UGT2B7	DAO	RORC	PDE4A	
CYP19A1	MAOB	PDE4D	RBP4	
SERPINA6	EGLN3	ALOX12	BCHE	
SHBG	GRIK1	SRD5A2	IMPDH2	
HSD17B3	GRIK2	SLC22A12	MMP2	
G6PD	GRM1	PGR	MCL1	
GABBR1	SCD	NOS2	BCL2	
PTGER2	PTGDR	PLA2G1B	PRKAG1	
PTGFR	PTGDR2	PTGER1	SLC6A3	
FNTA				
FNTB	TRPM8	RXRB	BACE1	
NPC1L1	CES1	PTGIR	CHRM2	
GABRA2	CES2	ADORA3	ACHE	
HAO1	SLC16A1	PTPRF	SLC6A2	
KDM2A	CPA3	TRPV1	SLC6A4	
HSD11B1	PTPN6	CD81	CYP2C19	
PLG	ENPEP	PRKCH	IDO1	
PTPN1	PTPN2	CYP26B1	RORA	
GSTK1	GRM3	CYP26A1	PDE4B	
LTA4H	GRM6	CYP51A1	GABRA2	
AKR1B10	EGLN1	ACP1	FNTA	
CA2	FOLH1	PTGER4	NR0B2	
CA1	CA12	NR1H3	RXRA	
CDC45	ESR1	OXER1	RARG	
PTPRC	ESR2	FFAR4	RARB	
CACNA2D1	CDC25B	NR3C2	RARA	
SLC6A12	NR1I3	SIGMAR1	RXRG	
PEPD	ANPEP	CYP17A1	ALOX15	

Table S2: Skin antiaging overlapping genes

ESR2	NOS2
PPARG	AHR
PTGS2	PPARA
RARA	MAPK14
PREP	ESR1
SRD5A2	MDM2
MAPK1	SCD
TERT	VDR
MMP2	PEPD
IL6	PPARD
HSD11B1	RARG
FABP5	NR3C1
TRPV1	ACP1
PTPRC	CNR1
AR	NR1H3

Table S3: Hair growth overlapping genes

HSD11B2	PTGDR2	MAOB
PTPN1	NOS2	CYP51A1
CHRM2	POLB	MAOB
PTPN6	RARG	AHR
SHH	RBP4	GRM8
CMA1	MAPK14	
SLC6A4	RARA	
PGR	IL6	
PPARA	GLUL	
EDNRA	SLC6A3	
RORC	PHF8	
GRM1	ITGAL	
DAO	SLC1A2	
CYP26A1	CYP19A1	
ESR1	GRM2	
ACP1	PTGES	
ANPEP	PREP	
ALOX5	PSEN2	
SAE1	ABCC1	
RARB	TERT	
PLG	TOP1	
NR3C2	HSD11B1	
RORA	KDM5C	
TOP2A	PTPN11	
CYP2C19	CYP26B1	
ABCB1	ALOX12	
MDM2	ESR2	
CES1	MAOA	
PDE4A	AR	
GSTK1	G6PD	
PPARG	VDR	
CHRNA7	CTSG	
TRPM8	BCHE	
SIGMAR1	CD81	
PTPRC	RXRA	
HMGCR	BCL2	
PTGFR	BACE1	
ACHE	MMP2	
SHBG	PTPN2	
PTGS2	CYP17A1	
TRPV1	PPARD	
SRD5A2	PTGS1	
MAPK3	MAPK1	
PTGER4	CDC45	
CPT1A	FABP3	
